# Development of glycinergic innervation to the murine LSO and SPN in the presence and absence of the MNTB

**DOI:** 10.3389/fncir.2014.00109

**Published:** 2014-09-12

**Authors:** Stefanie C. Altieri, Tianna Zhao, Walid Jalabi, Stephen M. Maricich

**Affiliations:** ^1^Richard King Mellon Foundation Institute for Pediatric Research and Department of Pediatrics, University of PittsburghPittsburgh, PA, USA; ^2^Department of Otolaryngology, University of PittsburghPittsburgh, PA, USA; ^3^Department of Pediatrics, Case Western Reserve UniversityCleveland, OH, USA

**Keywords:** hearing, deafness, mouse models, brain development, auditory system

## Abstract

Neurons in the superior olivary complex (SOC) integrate excitatory and inhibitory inputs to localize sounds in space. The majority of these inhibitory inputs have been thought to arise within the SOC from the medial nucleus of the trapezoid body (MNTB). However, recent work demonstrates that glycinergic innervation of the SOC persists in *Egr2*; *En1^CKO^* mice that lack MNTB neurons, suggesting that there are other sources of this innervation (Jalabi et al., 2013). To study the development of MNTB- and non-MNTB-derived glycinergic SOC innervation, we compared immunostaining patterns of glycine transporter 2 (GlyT2) at several postnatal ages in control and *Egr2*; *En1^CKO^* mice. GlyT2 immunostaining was present at birth (P0) in controls and reached adult levels by P7 in the superior paraolivary nucleus (SPN) and by P12 in the lateral superior olive (LSO). In *Egr2*; *En1^CKO^* mice, glycinergic innervation of the LSO developed at a similar rate but was delayed by one week in the SPN. Conversely, consistent reductions in the number of GlyT2^+^ boutons located on LSO somata were seen at all ages in *Egr2*; *En1^CKO^* mice, while these numbers reached control levels in the SPN by adulthood. Dendritic localization of GlyT2+ boutons was unaltered in both the LSO and SPN of adult *Egr2*; *En1^CKO^* mice. On the postsynaptic side, adult *Egr2*; *En1^CKO^* mice had reduced glycine receptor α1 (GlyRα1) expression in the LSO but normal levels in the SPN. GlyRα2 was not expressed by LSO or SPN neurons in either genotype. These findings contribute important information for understanding the development of MNTB- and non-MNTB-derived glycinergic pathways to the mouse SOC.

## INTRODUCTION

The pontine superior olivary complex (SOC) is the first central auditory region that receives significant bilateral acoustic information, and it is the primary site for detecting interaural level and timing differences (ILDs and ITDs) critical for sound localization. The neural pathways involved in these processes have been described in detail over the past few decades (Kandler et al., 2009). A critical player in both ILD and ITD detection is the medial nucleus of the trapezoid body (MNTB), which receives glutamatergic input from the contralateral cochlear nucleus (CN) and sends inhibitory projections to ipsilateral SOC nuclei. One major MNTB target is the lateral superior olive (LSO), which participates in ILD processing by integrating these inhibitory inputs with excitatory inputs from the ipsilateral CN (Boudreau and Tsuchitani, 1968; Moore and Caspary, 1983; Spangler et al., 1985; Sanes and Rubel, 1988; Helfert et al., 1989; Bledsoe et al., 1990; Wu and Kelly, 1991, 1992, 1995; Srinivasan et al., 2004). A second major target is the superior paraolivary nucleus (SPN), which also receives excitatory input from contralateral CN neurons (Friauf and Ostwald, 1988; Helfert et al., 1989; Thompson and Thompson, 1991; Schofield, 1995). The SPN encodes temporal sound features and has been proposed to function in sound localization, rhythm coding, gap detection and/or as a discontinuity detector (Behrend et al., 2002; Dehmel et al., 2002; Kulesza et al., 2003, 2007; Kadner et al., 2006; Kadner and Berrebi, 2008; Felix et al., 2014).

The primary neurotransmitter produced by the MNTB is glycine (Moore and Caspary, 1983; Peyret et al., 1987; Wenthold et al., 1987; Aoki et al., 1988; Helfert et al., 1989; Adams and Mugnaini, 1990). Studies done in many species demonstrate that glycinergic projections to SOC neurons develop during late embryogenesis and mature during the first two weeks of postnatal life, during which their action switches from depolarizing to hyperpolarizing (Wenthold et al., 1987; Helfert et al., 1989; Bledsoe et al., 1990; Sanes and Siverls, 1991; Kandler and Friauf, 1995; Kandler and Gillespie, 2005; Löhrke et al., 2005). In the rat, this switch is mirrored by the appearance and maturation of immunoreactivity for the glycine transporter type 2 (GlyT2; Friauf et al., 1999). Glycinergic network development in the SOC has not been well-studied in mice, where there is functional evidence for faster maturation of the auditory brainstem compared to other species (Ehret, 1976; Kullmann and Kandler, 2001). Understanding these pathways in mice is valuable given the wealth of genetic tools available to manipulate auditory regions for functional and behavioral investigations.

Recent work suggests that glycinergic projections to the SOC arise from additional sources other than the MNTB. Specifically, glycinergic innervation of the SOC is maintained in transgenic mice that lack MNTB neurons secondary to *Egr2^Cre^*-mediated conditional deletion of the *En1* gene in rhombomeres 3 and 5 (*Egr2*; *En1^CKO^* mice; Jalabi et al., 2013). These inhibitory projections are functional but exhibit prolonged inhibitory postsynaptic current (IPSC) decay time constants in both LSO and SPN neurons compared to those seen in control mice. The mechanisms that underlie these differences, as well as the development of these alternative glycinergic projections, have not been studied. Surprisingly, sound localization ability in *Egr2*; *En1^CKO^* mice is relatively preserved, suggesting that remarkable plasticity exists in the developing auditory brainstem.

In this study, we sought to characterize the postnatal development and localization of glycinergic inputs to the LSO and SPN using immunohistochemistry for GlyT2. We also analyzed expression patterns of glycine receptor subtypes Glyα1 and Glyα2 in adult control and *Egr2*; *En1^CKO^* mice to see if differences between the two might explain the altered IPSC decay kinetics. Our findings demonstrate that mice lacking MNTB neurons have alterations in the time course of somatic glycinergic innervation in the SPN and in the amount of somatic innervation in the LSO, while dendritic localization of glycinergic terminals is unaltered in both regions. Furthermore, decreased expression of the adult glycine receptor isoform, GlyRα1, was found in the LSO but not SPN of *Egr2*; *En1^CKO^* mice.

## MATERIALS AND METHODS

### ANIMALS

*Egr2^Cre/+^* mice (Voiculescu et al., 2000) on a C57BL/6J background were mated with *En1^flox/flox^* mice (Sgaier et al., 2007) on a mixed background to produce mice of four genotypes: *Egr2^+/+^*; *En1^+/flox^*, *Egr2^+/+^*; *En1^flox/flox^*, *Egr2^Cre/+^*; *En1^+/flox^* and *Egr2^Cre/+^*; *En1^flox/flox^* (*Egr2*; *En1^CKO^*; Jalabi et al., 2013). As no differences were seen previously between the control genotypes, we used only *Egr2^+/+^*; *En1^flox/flox^* mice as controls in this study. Brother-sister matings of *Egr2^+/+^*; *En1^flox/flox^* mice and *Egr2^Cre/+^*; *En1^flox/flox^* mice were used to produce offspring. Postnatal day 0 (P0, day of birth), P3, P7, P12, and P14 mice of both sexes were used for early postnatal experiments, while 8–10 month-old female mice were used for adult experiments (*N* = 2 mice/genotype at each age). Mice were maintained and housed on a 12:12 light:dark cycle with access to food and water *ad libitum*. All procedures were approved by the Case Western Reserve University and University of Pittsburgh Institutional Animal Care and Use Committees.

### TISSUE HARVESTING AND PROCESSING

Mice were deeply anesthetized with 300 mg/kg Avertin and transcardially perfused with ice-cold 4% paraformaldehyde (PFA). Whole brains were removed and post-fixed overnight at 4^∘^C in 4% PFA. Following post-fixation, brains were cryoprotected in 30% sucrose for 48 h. Brains were then embedded in Tissue-Tek O.C.T. compound (Sakura Finetek, Torrance, CA, USA), quickly frozen and stored at -80^∘^C before sectioning. Brains were sectioned in the coronal plane at 10 μm thickness using a CM1950 cryostat (Leica, Buffalo Grove, IL, USA). Sections were collected onto Superfrost/Plus slides (Thermo Fisher Scientific), dried overnight and stored in -80^∘^C prior to use. Slide sets with 50 μm (adult) or 30 μm (young postnatal) separation between sections were prepared to allow systematic sampling through the SOC, and each set was immunostained with a different antibody

### IMMUNOHISTOCHEMISTRY

Slides containing sections through the SOC were rehydrated in 1× PBS for 5 min and then incubated in blocking solution (0.3% Triton X-100, 3% donkey serum in 1× PBS) for 1 h. Primary antibodies were diluted in blocking solution and incubated overnight at 4^∘^C. The following primary antibodies and concentrations were used: guinea-pig anti-glycine transporter 2 (GlyT2) polyclonal antibody (Millipore, Temecula, CA) at 1:1000; chicken anti-MAP2 polyclonal antibody (Abcam Inc., Cambridge, MA, USA) at 1:10000; rabbit anti-glycine receptor alpha 1 (GlyRα1) polyclonal antibody (Millipore, Temecula, CA, USA) at 1:1000; goat anti-glycine receptor alpha 2 (GlyRα2) polyclonal antibody at 1:500 (Santa Cruz Biotechnology, Dallas,TX). Slides were washed 3 × 5 min in 1× PBS. Secondary antibodies were diluted in blocking solution at 1:500 and incubated on the slides for 1 h at room temperature. The following secondary antibodies were used: Alexa Fluor 594-AffiniPure donkey anti-guinea pig, Alexa Fluor 488-AffiniPure donkey anti-chicken, Alexa Fluor 488-AffiniPure donkey anti-rabbit and Alexa Fluor 488-AffiniPure donkey anti-goat (Jackson ImmunoResearch, West Grove, PA). All slides were counterstained with DAPI. Following secondary antibody incubation, slides were washed 3 × 5 min in 1× PBS. In some cases, slides were counterstained with NeuroTrace Green Fluorescent Nissl (Life Technologies, Grand Island, NY) at a concentration of 1:100 diluted in 1× PBS for 30 min at room temperature. Slides were washed an additional 3 × 5 min in 1× PBS and then mounted with ProLong Gold (Life Technologies, Grand Island, NY). Slides were imaged using a Leica DM5500B epifluorescence microscope or an inverted Zeiss Axio Observer on a PerkinElmer Ultra*VIEW* VoX spinning disk confocal with a Hamamatsu C9100-13 camera and Volocity software.

### BOUTON COUNTS AND LOCALIZATION

GlyT2+ boutons located on LSO and SPN neuron somata of P7, P12 and P14 mice were counted on six sections/side (*N* = 12 sections/mouse) on photographs taken at 60 μm increments on a Leica DM5500B epifluorescence microscope. For adult mice, bilateral LSO and SPN images across 10 sections/side (*N* = 20 sections/mouse) at 50 μm increments were used for counting. Images were processed using ImageJ 1.47V (NIH), and the number of boutons on all neuronal soma with an identifiable nucleus were counted and reported as mean puncta per neuron ± SEM. For analyzing localization of glycinergic innervation in the LSO or SPN, boutons were measured by distance from the cell body using Volocity software. The origin of the dendrite (0 μm distance from the soma) was defined as the region where the soma narrowed to form a distinct process. Neurons with an identifiable nucleus and visible dendrites at least 10 μm in length were used for analysis (*N* = 12 dendrites/genotype/brain region). Glycine receptor α1 isoform (GlyRα1) clusters were quantified by counting the numbers of immunopositive puncta located on LSO or SPN neuronal somata (*N* = 4 sections/mouse at 50 μm increments).

### STATISTICAL ANALYSIS

The number of boutons on LSO or SPN neuronal somata were compared using non-parametric Mann–Whitney *U* tests for genotype comparisons at the same age and Kruskal–Wallis tests with Dunn’s multiple comparisons for age effects within a genotype. The number of boutons located in 5 μm segments up to 15 μm from the cell soma and the number of GlyRα1+ puncta were compared using Mann–Whitney tests. All statistical analyses were performed using GraphPad Prism software (La Jolla, CA, USA).

## RESULTS

### DEVELOPMENT OF GLYCINERGIC PROJECTIONS TO THE SOC OF *Egr2*; *En1^CKO^* MICE AND CONTROL LITTERMATES DIFFERS IN TEMPORAL PATTERNING AND QUANTITY

**FIGURE 1:**
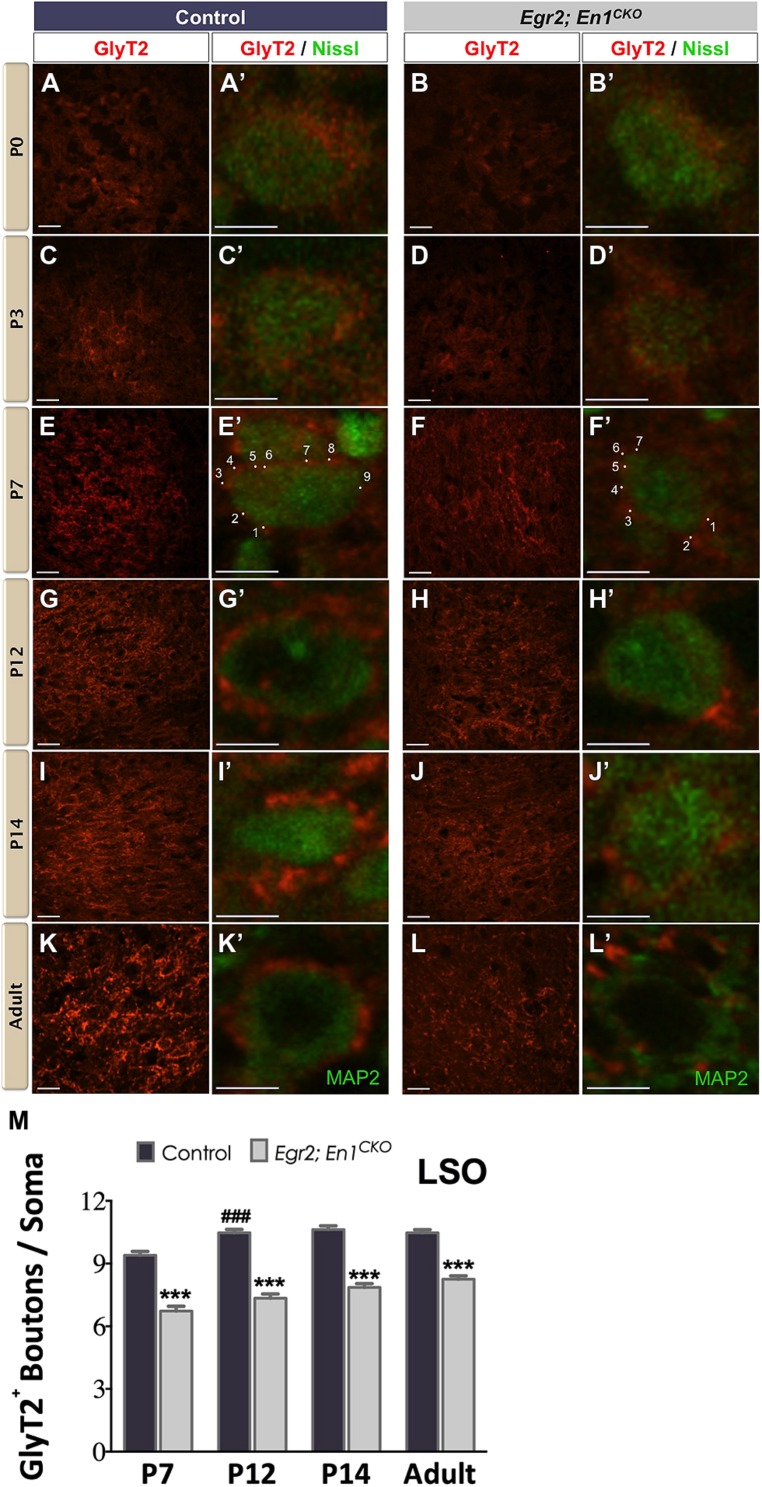
**Development of glycinergic innervation to the LSO of control and *Egr2*; *En1^**CKO**^* mice.** Diffuse GlyT2 immunoreactivity was detected as early as P0 in control **(A,A’)** and *Egr2*; *En1^CKO^* mice **(B,B’)**. Slight increases in staining intensity were seen in P3 control **(C,C’)** and *Egr2*; *En1^CKO^* mice **(D,D’)**. By P7, boutons located on LSO neuronal somata were easily discernible in both control **(E,E’)** and mutant mice **(F,F’),** albeit to a lesser extent in the latter. Individual boutons are shown with dots and numbers. By P12, levels of glycinergic innervation were increased in control mice **(G,G’)** but remained constant in *Egr2*; *En1^CKO^* mice **(H,H’)** compared to P7. Compared to controls **(I,I’,K,K’)**, *Egr2*; *En1^CKO^* mice had reduced glycinergic innervation at two weeks of age and in adulthood **(J,J’, L,L’)**. The number of GlyT2^+^ boutons was counted in P7 and older mice when boutons were easily recognizable. Reductions in somatic GlyT2+ bouton number occurred in *Egr2*; *En1^CKO^* mice compared to control littermates at all ages examined **(M)**. Data are represented as mean ± SEM. Higher magnification images of individual neurons are shown in **(A’–L’)**. Scale bars: 20 μm **(A–D)**; 26 μm **(E–L)**; 8 μm **(A’–L’)**. ***P < 0.001 vs. age-matched controls, ^###^*P* < 0.001 vs. genotype-matched P7 mice.

We immunostained for GlyT2 to label glycinergic projections (Friauf et al., 1999) to the LSO and SPN throughout postnatal development starting on the day of birth (P0). In agreement with previous reports (Jursky and Nelson, 1996; Friauf et al., 1999), diffuse GlyT2 immunoreactivity was present in the LSO of littermate control and *Egr2*; *En1^CKO^* mice at P0 and P3 (**Figures 1A–D**′). By P7, distinct boutons were present on cell somata (**Figures 1E–F**′). The number of boutons/soma increased between P7 and P12 in controls (*P* < 0.04) then remained constant at P14 and in adulthood (9.40 ± 0.19 vs. 10.47 ± 0.16 vs. 10.63 ± 0.17 vs. 10.47 ± 0.15, respectively; *N* = 57–168 soma from 12 to 20 LSO sections/mouse, *N* = 2 mice/age; *P* < 0.001 for age effects). The numbers of GlyT2+ boutons/soma also increased with age in *Egr2*; *En1^CKO^* mice (*P* < 0.001), but were consistently 20–30% lower than control values at P7, P12, P14, and adulthood (6.73 ± 0.23 vs. 7.35 ± 0.20 vs. 7.86 ± 0.18 vs. 8.25 ± 0.16; *N* = 39–102 soma from 12 to 20 LSO sections/mouse, *N* = 2 mice/age; **Figures 1E–M**). Thus, glycinergic innervation to the LSO in *Egr2*; *En1^CKO^* mice developed following a similar time course to that seen in littermate controls, but never reached control levels.

**FIGURE 2:**
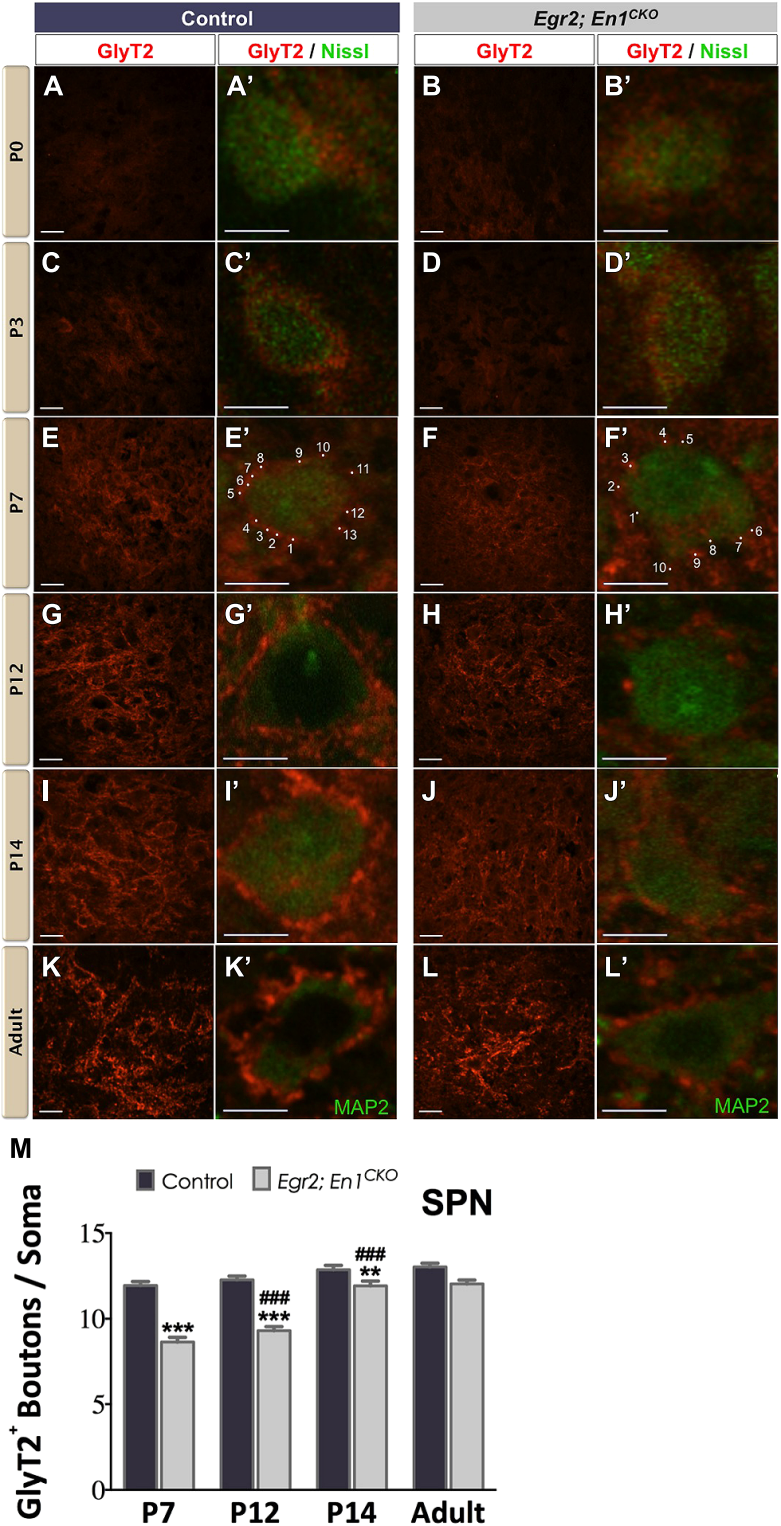
**Development of glycinergic innervation to the SPN of control and *Egr2*; *En1^**CKO**^* mice.** Diffuse GlyT2 staining was present at birth in control **(A,A’)** and *Egr2*; *En1^CKO^* mice **(B,B’)**. Staining intensity increased by P3 in control **(C,C’)** and *Egr2*; *En1^CKO^* mice **(D,D’)**. Identifiable boutons were first seen at P7 in control **(E,E’)** and *Egr2*; *En1^CKO^*** (F,F’)** mice. Individual boutons are shown with dots and numbers. Staining intensity was also higher in control **(G,G’)** compared to *Egr2*; *En1^CKO^* mice **(H,H’)** at P12. However, levels were comparable in control and *Egr2*; *En1^CKO^* mice at P14 **(I,I’,J,J’)** and adulthood **(K,K’,L,L’)**. **(M)** GlyT2+ bouton number located on SPN neuronal somata did not change from P7-adulthood in control mice. Bouton numbers were reduced at from P7–P14 in *Egr2*; *En1^CKO^* mice, but were similar to controls in adulthood. Data are represented as mean ± SEM. Higher magnification images of individual neurons are shown in **(A’–L’)**. Scale bars: 20 μm **(A–D)**; 26 μm **(E–L)**; 8 μm **(A’–L’)**. **P < 0.01, ***P < 0.001 vs. age-matched controls and ^###^*P* < 0.001 vs. P7 or P12 *Egr2*; *En1^CKO^* mice.

Similar to the LSO, diffuse GlyT2+ labeling was present in the SPN of P0 and P3 littermate controls and *Egr2*; *En1^CKO^* mice (**Figures 2A–D**′). In control mice, the number of boutons/soma remained constant at P7, P12, P14, and adulthood, suggesting that maturation was achieved by the end of the first postnatal week (11.94 ± 0.23, vs. 12.27 ± 0.22 vs. 12.86 ± 0.25 vs. 13.02 ± 0.22, respectively, *N* = 49–81 soma from 12 to 20 SPN sections/mouse, *N* = 2 mice/age; *P* = 0.06 for age effects). In *Egr2*; *En1^CKO^* mice, the number of boutons/soma gradually increased from P7 through P14 when numbers were similar to adults (8.63 ± 0.27, 9.30 ± 0.24, 11.92 ± 0.27, 12.03 ± 0.22, respectively, *N* = 36–81 soma from 12 to 20 SPN sections/mouse, *N* = 2 mice/age; *P* < 0.001 for age effects). While the numbers of boutons/soma were reduced by 25–30% compared to littermate controls at P7–P14, no differences were detected in adults, suggesting that development of glycinergic inputs to the SPN is simply delayed in *Egr2*; *En1^CKO^* mice (**Figures 2E–M**).

### DENDRITIC LOCALIZATION OF GLYCINERGIC INNERVATION IS UNALTERED IN *Egr2*; *En1^CKO^* MICE

**FIGURE 3:**
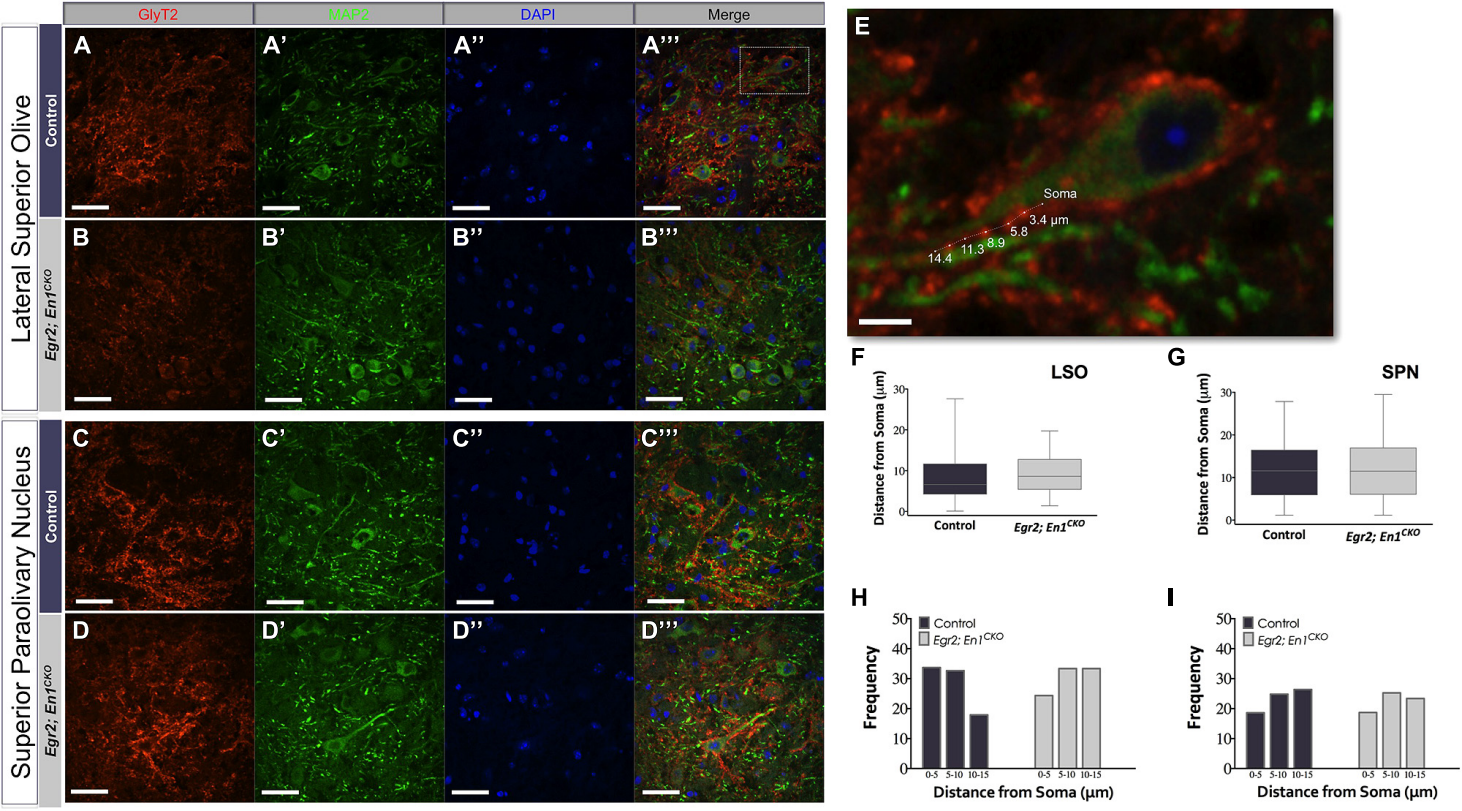
**Dendritic localization of GlyT2^+^ boutons is unchanged in adult *Egr2*; *En1*^**CKO**^ mice.** GlyT2+ boutons were present on MAP2+ dendrites of LSO and SPN neurons in adult control **(A–A″′,C–C″′)** and *Egr2*; *En1^CKO^* mice **(B**–**B″′,D**–**D″′)**. **(E)** Area outlined by the white box in **(A″′)**. Distance of GlyT2^+^ boutons from neuronal soma was measured as shown. Box plots depicting the median and distribution of bouton distance from cell somata in LSO **(F)** and SPN **(G)**. Whiskers are representative of minimum and maximum distance values. Data were further analyzed by looking at proximal vs. distal localization by determining the frequency (percentage) of boutons in 5 μm increments for dendrites in the LSO **(H)** and SPN **(I)**; no significant differences were present between control and *Egr2*; *En1^CKO^* mice. Scale bars: 33 μm **(A–D″′)**; 16 μm **(E)**.

We next examined whether *Egr2*; *En1^CKO^* mice exhibited alterations in glycinergic innervation of LSO and SPN neuron dendrites by co-immunostaining for GlyT2 and microtubule-associated protein 2 (MAP2; **Figures 3A–D′′′**). The overall distribution of boutons along the proximal 15 μm of LSO and SPN neuron dendrites was similar in littermate control and *Egr2*; *En1^CKO^* mice (**Figures 3E–G**). Furthermore, the number of boutons binned into 5 μm segments up to 15 μm from the cell soma was also similar (LSO – Control: 0–5 μm – 2.67 ± 0.47, 5–10 μm – 2.58 ± 0.36, and 10–15 μm – 1.67 ± 0.31; *Egr2*; *En1^CKO^* mice: 0–5 μm – 1.58 ± 0.40, 5–10 μm – 2.17 ± 0.42, and 10–15 μm – 2.17 ±0.27; *P* = 0.10, *P* = 0.65, and *P* = 0.26, respectively and SPN – Control: 0–5 μm – 2.00 ± 0.35, 5–10 μm – 2.67 ± 0.36, and 10–15 μm – 2.92 ± 0.29; *Egr2*; *En1^CKO^* mice: 0–5 μm – 1.82 ± 0.42, 5–10 μm – 2.46 ± 0.28, and 10–15 μm – 2.27 ± 0.36; *P* = 0.50, *P* = 0.87, and *P* = 0.13, respectively; **Figures 3H,I** insets and data not shown). Taken together, these data indicate that the number and location of dendritic GlyT2^+^ boutons is similar in control and *Egr2*; *En1^CKO^* mice.

### *Egr2*; *En1^CKO^* MICE HAVE REDUCED SOMATIC EXPRESSION OF THE GlyRα1 GLYCINE RECEPTOR SUBUNIT IN LSO BUT NOT SPN NEURONS

**FIGURE 4:**
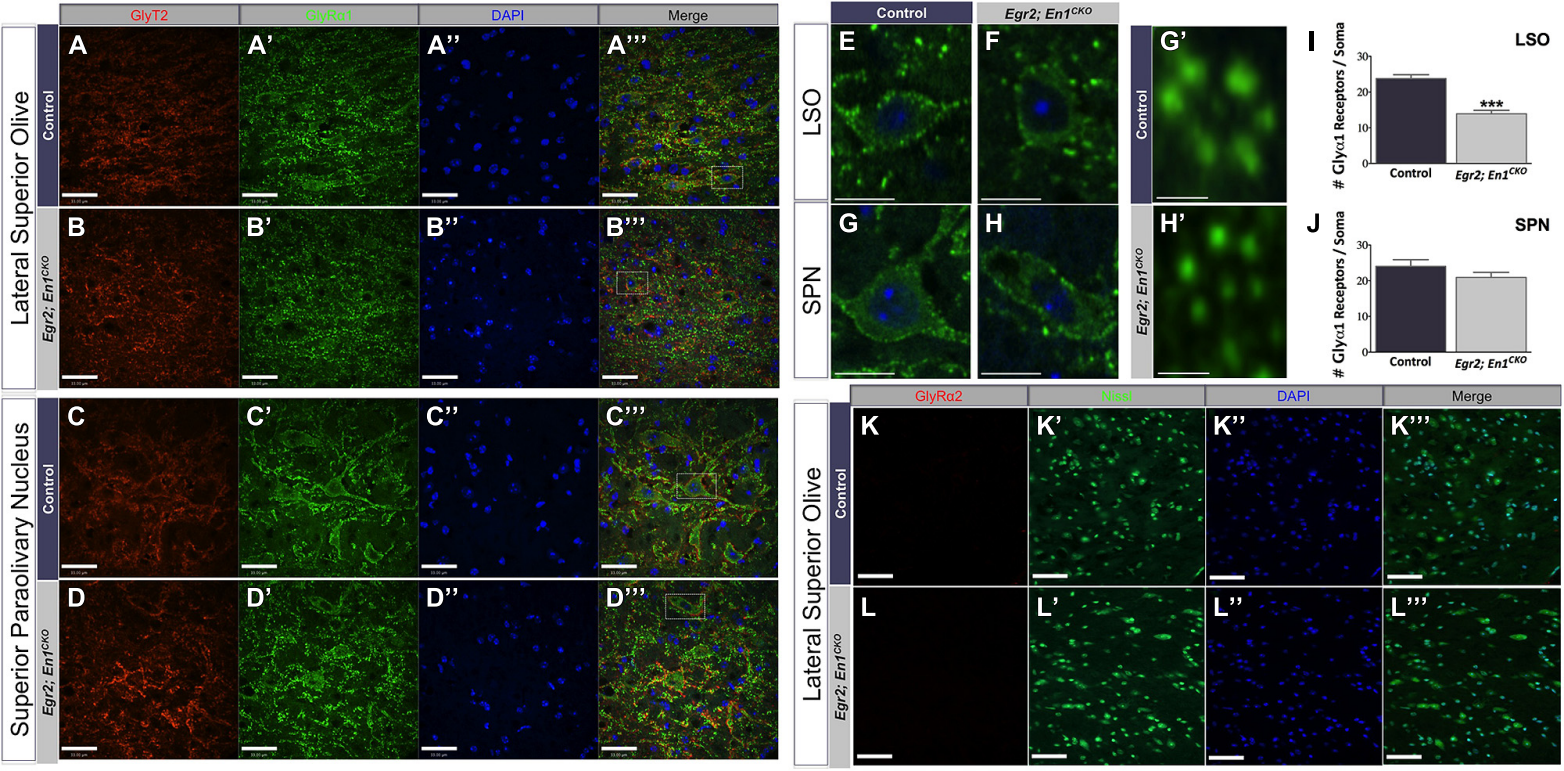
**Glycine receptor isoform expression patterns, but not receptor number, are similar in LSO and SPN neurons of adult control and *Egr2*; *En1^**CKO**^* mice.** Adult control and *Egr2*; *En1^CKO^* mouse brain sections through the LSO **(A,B)** and SPN **(C,D)** were immunostained for GlyT2 **(A–D)** and the glycine receptor isoform alpha 1 (GlyRα1; **A’–D’**). **(E–H)** Higher magnification images showing punctate staining on LSO **(E,F)** and SPN **(G,H)** neuronal somata and dendrites are shown in control **(E,G)** and *Egr2*; *En1^CKO^* **(F,H)** mice. **(G’,H’)** High magnification images of typical GlyRα1 rosettes. Counts of GlyRα1+ puncta per LSO **(I)** or SPN **(J)** neuronal soma revealed decreased numbers in the LSO, but not SPN, of *Egr2*; *En1^CKO^* compared to control mice. Data are represented as mean ± SEM. Glycine receptor alpha 2 (GlyRα2) was not expressed by LSO neurons in adult control **(K)** or *Egr2*; *En1^CKO^* mice **(L)**. DAPI **(A–D”,K”,L”)** and Nissl **(K’,L’)** counterstains are also shown. Scale bars: 33 μm **(A–D,K,L)**; 12 μm **(E–H)**; 2.3 μm **(G’,H’)**. ***P < 0.001 vs. control.

We previously reported that glycinergic IPSC decay time constants were >2.5-fold slower in LSO and SPN neurons of *Egr2*; *En1^CKO^* mice compared to littermate controls (Jalabi et al., 2013). We hypothesized that differences in the number or subunit composition of glycine receptors expressed by LSO and SPN neurons might explain these differences. Therefore, we examined expression patterns of two well-characterized glycine receptor subtypes, the adult-like isoform (GlyRα1) and the purported developmental isoform (GlyRα2). Punctate GlyRα1 immunoreactivity was present in LSO and SPN neurons of both genotypes (**Figures 4A–H**). However, the number of GlyRα1+ clusters/soma was significantly higher in LSO neurons of control vs. *Egr2*; *En1^CKO^* mice (23.78 ± 1.07 vs. 13.91 ± 0.97; *N* = 21–29 soma from 4 nuclei/mouse, *N* = 2 mice/genotype, *P* < 0.001; **Figure 4I**). No difference was found in SPN neurons (control: 24.03 ± 1.85 vs. *Egr2*; *En1^CKO^* mice: 20.93 ± 1.41; *N* = 17–27 soma from four nuclei/mouse, *N* = 2 mice/genotype, *P* = 0.08; **Figure 4J**). In both control and *Egr2*; *En1^CKO^* mice, typical GlyRα1 rosettes (Hruskova et al., 2012) could be identified and glycinergic receptors were apposed to GlyT2_+_ boutons (**Figures 4A″′,B″′,C″′,D″′,G″′,H″′**). GlyRα2 expression was not present in LSO (**Figures 4K–L″′**) or SPN (data not shown) neurons in control or *Egr2*; *En1^CKO^* mice. This suggests that a failure to switch to the adult isoform (fast decay kinetics) from the developmental isoform (slower decay kinetics) does not account for altered IPSC kinetics in these mice.

## DISCUSSION

Our data demonstrate that glycinergic innervation measured by GlyT2 immunoreactivity in the mouse LSO and SPN follows a similar developmental time course to that seen in other rodents such as guinea pigs and rats (Wenthold et al., 1987; Helfert et al., 1989, 1992; Friauf et al., 1999). Gradual increases in GlyT2 expression and the formation of distinct somatic boutons during the first postnatal week coincide with the timeframe of synaptic pruning and strengthening of connections arising from the MNTB during the transient excitatory period of these developing networks (Kim and Kandler, 2003). Adult-like GlyT2 expression occurs sooner in the SPN than in the LSO, and both mature in advance of hearing onset (Willott, 2001). This pattern coincides with the earlier functional maturation of SPN neurons demonstrated by their earlier switch from depolarizing (excitatory) to hyperpolarizing (inhibitory) glycinergic post-synaptic responses, which occurs by birth in the SPN but not until P3–P5 in the LSO (Kullmann and Kandler, 2001; Löhrke et al., 2005). One difference between rats and mice is that the intensity of GlyT2 immunoreactivity reaches maximum levels at P10 in the rat (Friauf et al., 1999), while in mice it is maximal in the adult (**Figures 1K–L″′ and 2K–L″′**).

Our data provide insights into the development of glycinergic SOC innervation in the absence of MNTB neurons. In the LSO, the number of GlyT2+ boutons/soma increases at a similar rate from P7 to adulthood but is consistently 20–30% lower in *Egr2*; *En1^CKO^* than controls. This suggests that the developmental time course of GlyT2 immunoreactivity is similar in both genotypes. Conversely, glycinergic development is delayed in the SPN, as the number of glycinergic boutons/SPN neuron soma does not reach adult levels until P14 in *Egr2*; *En1^CKO^* mice, 7 days after controls. However, the number of boutons/soma is similar in adult *Egr2*; *En1^CKO^* and littermate control mice, suggesting that development is simply delayed rather than permanently altered as it is in the LSO. GlyT2 function is theorized to influence the development of inhibitory networks in the SOC because its expression precedes synapse maturation in multiple central auditory regions (Kandler and Friauf, 1995; Ehrlich et al., 1998; Friauf et al., 1999). Thus, reduced levels of GlyT2 expression in the LSO and SPN of *Egr2*; *En1^CKO^* mice during early postnatal development could contribute to persistent changes in glycinergic circuitry and function in these mice.

The number and distribution of GlyT2+ boutons on the dendrites of LSO and SPN neurons is similar in adult *Egr2*; *En1^CKO^* mice and littermate controls (**Figure 3**). This is consistent with the interpretation that non-MNTB-derived glycinergic projections normally target the dendrites, and ectopically expands to LSO and SPN neuronal somata only in the absence of competition from MNTB-derived projections. Alternatively, this could occur if improper refinement and/or synaptic pruning of distally located boutons takes place in the absence of MNTB-derived projections. For example, developmentally regulated activity-dependent relocation of inhibitory inputs from dendrites to cell somata occurs in medial superior olive (MSO) neurons of animals with well-developed low frequency hearing (Kapfer et al., 2002); whether a similar process occurs in the LSO of animals that rely on high-frequency hearing (like the mouse) is not known. A third possibility is that MNTB- and non-MNTB-derived glycinergic inputs normally intermingle on cell somata and dendrites, and that the non-MNTB-derived innervation expands in both locations in the absence of MNTB neurons. Identification of the source of the non-MNTB-derived innervation followed by labeling of its projections is necessary to distinguish between these possibilities. Regardless, changes in glycinergic bouton localization cannot cause the altered IPSC kinetics seen in LSO and SPN neurons of *Egr2*; *En1^CKO^* mice (Jalabi et al., 2013).

We also examined GlyR subunit expression in the LSO and SPN of adult control and *Egr2*; *En1^CKO^* mice. Glycine receptors are multimeric proteins composed of 2 alpha and 3 beta subunits (Lynch, 2009; Dutertre et al., 2012; Yang et al., 2012). Alpha subunits contain the ligand binding pocket and come in four isoforms (GlyRα1-α4), each of which confers different functional channel properties (Pfeiffer et al., 1982). We focused on GlyRα1 and GlyRα2 because these receptors are expressed by developing and/or mature SOC neurons of other species (Friauf et al., 1997; Piechotta et al., 2001), and they confer fast and slow IPSC decay kinetics to the GlyR, respectively (Takahashi et al., 1992; Bormann et al., 1993; Singer et al., 1998; Weiss et al., 2008). Our finding that GlyRα1 was highly expressed by LSO and SPN neurons of adult mice agrees with previous studies in adult rats (Sato et al., 1995; Friauf et al., 1997; Piechotta et al., 2001). GlyRα1 expression was detected in the LSO of *Egr2*; *En1^CKO^* mice, but the number of receptor clusters per neuronal cell body was reduced compared to controls. This reduction, along with the reduced number of GlyT2+ boutons, may contribute to the decreased IPSC amplitudes seen in LSO neurons of these mice (Jalabi et al., 2013). Our hypothesis that maintained expression of GlyRα2, which is normally expressed only during embryonic and postnatal ages (Becker et al., 1988; Akagi et al., 1991; Malosio et al., 1991; Sato et al., 1995; Kungel and Friauf, 1997; Piechotta et al., 2001), might explain the aberrant IPSC decay kinetics proved to be incorrect as GlyRα2 was not expressed by LSO or SPN neurons in adult control or *Egr2*; *En1^CKO^* mice. We did not examine GlyRα4 because it is minimally expressed in the CNS (Harvey et al., 2000) and therefore is unlikely to contribute to the altered electrophysiological responses seen in *Egr2*; *En1^CKO^* mice. Similarly, we did not examine β subunit distribution as alterations in its expression are predicted to affect glycine receptor trafficking and clustering (Kneussel and Betz, 2000), which appear to be unaffected in *Egr2*; *En1^CKO^* mice (**Figures 4E″′,F″′**). Future studies could examine GlyRα3 expression, which shows similar kinetics to GlyRα1 and is expressed in the auditory brainstem, albeit at lower levels (Sato et al., 1995).

Taken together, our findings demonstrate dynamic developmental reprogramming of the SOC glycinergic circuitry in the absence of the MNTB. Identifying the source(s) of this non-MNTB-derived innervation in *Egr2*; *En1^CKO^* mice in conjunction with experiments to measure function and expression of receptors at critical developmental time points is necessary to clarify factors that lead to persistent changes. The striking ability of this system to compensate in the absence of the MNTB has potential clinical implications that might be important for understanding altered central auditory function in autism, where neuron number is lower in many SOC subnuclei (Rosenblum et al., 1980; Wong and Wong, 1991; Maziade et al., 2000; Kulesza, 2008; Kulesza et al., 2011), and in age-related hearing loss (Grimsley and Sivaramakrishnan, 2014).

## Conflict of Interest Statement

The authors declare that the research was conducted in the absence of any commercial or financial relationships that could be construed as a potential conflict of interest.
